# Workplace violence among nursing professionals

**DOI:** 10.47626/1679-4435-2020-531

**Published:** 2021-02-11

**Authors:** Maria Luiza Guidinho Bernardes, Marcia Eiko Karino, Júlia Trevisan Martins, Caroline Vieira Cláudio Okubo, Maria José Quina Galdino, Aline Aparecida Oliveira Moreira

**Affiliations:** 1 Centro de Ciências da Saúde, Universidade Estadual de Londrina, Londrina, PR, Brazil; 2 Departamento Enfermagem, Universidade Estadual do Norte do Paraná, Bandeirantes, PR, Brazil

**Keywords:** occupational health, nursing staff, emergency hospital services, workplace violence

## Abstract

**Introduction:**

The frequency of workplace violence has increased significantly across several countries, with short- and long-term effects on workers’ health. Within the health care sector, nursing professionals are the most exposed to workplace violence, since they provide direct assistance to patients on a 24-hour basis.

**Objectives:**

To identify the types of occupational violence experienced by nursing professionals.

**Methods:**

This was a descriptive, quantitative study of 55 nursing professionals in the emergency unit of a public hospital. Data were collected between April and June 2018 through the Questionnaire for Workplace Violence Experienced or Witnessed by Nursing Professionals. Categorical variables were presented as frequencies and percentages, while continuous variables were described using measures of central tendency and dispersion.

**Results:**

A total of 49 (88.9%) participants had experienced workplace violence, with 21 (38%) suffering verbal abuse; 14 (25.4%) experiencing mobbing; 6 (11%) reporting physical violence; 5 (9.1%) suffering sexual harassment; and 3 (5.4%) reporting racial discrimination. Furthermore, 44 (90%) of these individuals reported that the incidents of violence could have been prevented.

**Conclusions:**

The types of violence observed included physical aggression, verbal abuse, sexual harassment and racial discrimination, with verbal abuse being the most commonly reported. These acts were perpetrated by patients and their families, as well as colleagues and supervisors. Violence prevention strategies should be implemented in institutions in order to provide a safe working environment.

## INTRODUCTION

Workplace violence is a global public health concern of growing magnitude, which has been increasingly studied due to its immediate and long-term impact on worker health.^1^ There is no consensus on the definition of workplace violence, but in the present study, this was understood as a multifactorial concept, arising from the interplay of individual, relational, cultural, and environmental factors, and resulting in an action, incident or behavior that departs from conventional expectations and causes a worker to be threatened or harmed in the course of, or as a direct consequence of, their occupation.^2^

Workplace violence can be classified according to its structure as either vertical or horizontal. Vertical violence involves both health care workers and patients, while horizontal violence occurs exclusively between health care workers or patients themselves.^3^ Incidents can also be classified according to type, as manifestations of either physical or psychological violence. Physical violence includes assaults, beatings, spitting, kicking or even homicide. Psychological violence, on the other hand, can be further divided into verbal abuse, bullying, sexual harassment and racial discrimination, and includes acts of intimidation, coercion, defamation, slander, blackmail, verbal and non-verbal threats, verbal and non-verbal abuse, mobbing and sexual harassment.^2,4^ In the present study, verbal violence was defined as the use of language (insults, swearing or yelling) to inflict harm while mobbing was defined as the result of slander, defamation or libel.

While violence affects workers in all occupations, it is thought to be especially prevalent in the health care sector due to occupational characteristics including, but not limited to, long hours; shift work; frequent contact with death; and the lack of human resources, material and equipment.^2^ The health care sectors where workers are thought to have the highest risk of exposure to violence are psychiatric, geriatric and emergency departments.^5^ It is also important to note that working directly with people who have a history of violence and drug or alcohol abuse, in poorly-lit environments with little to no safety and in regions with high crime rates are all risk factors for exposure to workplace violence by patients and their families.^5^

Nurses represent a significant proportion of the health care workforce, and provide 24-hour care to patients on a continuous basis, spending most of their working time in direct contact with patients; as a result of these factors, they are especially vulnerable to occupational violence.^6^ A study of 447 nurses in two hospitals in Amman and Jordan found that the prevalence rates of verbal and physical abuse in this population were 37.1 and 18.3%, respectively. Less experienced nurses and those working in emergency and intensive care units reported the highest rates of violence. Furthermore, over half the nurses who experienced abuse considered leaving their job, and reported a decrease in work-related quality of life.^7^

Brazilian studies^8,9^ have found that health care professionals are highly exposed to workplace violence, with nurses being the most likely to experience these events. A study of 8,345 nursing professionals in Brazil found that 19.7% of them had experienced some form of violence, with psychological, physical and sexual aggression accounting for 66.5, 15.6 and 0.9% of these cases, respectively. Among male nurses, psychological (65.2%) violence was most prevalent, followed by physical (10.6%) and sexual (0.6%) aggressions.^10^ Exposure to occupational violence is associated with health issues such as anxiety, insomnia,^11^ and symptoms of burnout*,*^12^ which can lead to workplace absence and even death.^13^

In light of these observations and the scarcity of studies on the topic in the Brazilian literature, the present study aimed to identify the types of workplace violence experienced by nursing professionals.

## METHOD

This was a descriptive, quantitative study of the nursing staff in the emergency unit of a teaching hospital in southern Brazil. The hospital in question is a public institution and a reference for high-complexity treatments, whose 313 beds are all designated to patients in the Unified Health Service (SUS). Data were collected by convenience, and the emergency ward was selected as the study site due to the high risk of workplace violence observed in these settings. This study involved 55 nurses from all shifts, assessed between April and June 2018. Inclusion criteria consisted of working in the emergency unit for at least 1 year, regardless of contract type (public servant or private contractor), and not going on vacation or leave during the study period.

Data were collected using the Questionnaire for Workplace Violence Experienced or Witnessed by Nursing Professionals, developed and validated in 2005,^14^ based on the model proposed by the World Health Organization (WHO), International Labor Organization (ILO), Public Services International and International Council of Nurses.^15^ It is a self-report questionnaire with 54 items pertaining to the identification and description of incidents of physical violence, verbal abuse, sexual harassment, mobbing or racial discrimination experienced or witnessed by nurses.^14^ The items were divided into groupings based on the type of violence and preceded by the definition of the concept in question. The following sociodemographic and occupational variables were also investigated: race; gender; age; professional category; education level; length of nursing career; number of current jobs; weekly hours of work; perceived recognition at work; concern for workplace violence; and presence of procedures and encouragement for reporting violence in the workplace.

Each participant was given an opaque envelope containing the questionnaire. At this point, participants were informed of the aims of the study and asked to return the envelopes to the researcher responsible for the study on the same day or the following shift. Participants were also told to answer the questionnaires based on their work in the emergency room, even if they had other jobs. Statistical analyses were performed using Epi Info, version 2016, developed by the *Centers for Disease Control and Prevention* (CDC). Categorical variables were presented as frequencies and percentages, while continuous variables were described using measures of central tendency and dispersion. For the analysis of workplace violence, patients were dichotomized into categories based on whether or not they had experienced physical violence, verbal abuse, sexual harassment, mobbing or racial discrimination.

This study was conducted according to ethical principles and approved by the Research Ethics Committee under project number 2.386.855. All participants provided written informed consent to taking part in the study.

## RESULTS

The study involved 55 participants, 40 (72.7%) of whom were female. Thirty (54.5%) participants were in the 40-year age group and 39 (71%) identified as white. With regard to occupation, 32 (58%) participants were nursing technicians, 16 (29%) were nurses and 7 (13%) were nursing assistants. Questions regarding education showed that 20 (36.4%) workers completed secondary school and 20 (36.4%) had higher education qualifications. Additionally, 15 (27.2%) participants had over 26 years of nursing experience, 45 (82%) had only one job and 45 (81.8%) worked 36 hours a week. Most workers, 45 (82%), did not feel recognized for their work and 26 (47%) reported concerns about workplace violence. Questions regarding reports of workplace violence revealed that 38 (69%) participants claimed there were no standardized reporting procedures at their institution, and 46 (83.6%) felt no encouragement to report incidents of violence. Table 1 describes the different types of occupational violence identified by the nursing staff, 49 (88.9%) of whom had experienced some form of violence in the past.

**Table 1:** Occurrence of different types of workplace violence among nursing professionals, Londrina, PR, Brazil, 2018.

Incident	Type of violence				
	Physical	Verbal abuse	Mobbing	Sexual harassment	Racial discrimination
	n (%)	n (%)	n (%)	n (%)	n (%)
Yes	6 (11.0)	21 (38.0)	14 (25.4)	5 (9.1)	3 (5.4)
No	49 (89.0)	33 (60.0)	39 (71.0)	48 (87.3)	50 (91.0)
Missing	0 (0.0)	1 (2.0)	2 (3.6)	2 (3.6)	2 (3.6)
Total	55 (100.0)	55 (100.0)	55 (100.0)	55 (100.0)	55 (100.0)

Table 2 shows the frequency of each type of occupational violence among the 49 participants who experienced these events in the past. Women experienced the highest number of events across all categories, including physical or verbal abuse, bullying, sexual harassment and racial discrimination. Most incidents were perpetrated by patients and colleagues, and occurred within the institution.

**Table 2:** Occurrence of different types of workplace violence among nursing professionals, as categorized by gender, frequency, perpetrator, location and possibility of prevention, Londrina, PR, Brazil, 2018.

Type of violence	Physical	Verbal abuse	Mobbing	Sexual harassment	Racial discrimination	Total n (%)
Gender						
Male	0	5	2	2	1	10 (20.5)
Female	6	15	11	3	2	37 (75.5)
Missing	0	1	1	0	0	2 (4.0)
Frequency in the past 12 months						
All the time	0	0	2	0	0	2 (4.0)
Sometimes	0	5	9	3	3	20 (41.0)
Once	6	16	3	2	0	27 (55.0)
Perpetrator						
Patient/relatives of patient	6	7	3	2	2	20 (41.0)
Colleague/supervisor	0	14	11	3	1	29 (59.0)
Location of incident						
Inside institution	6	20	13	5	3	47 (96.0)
Outside (on the way to/from work)	0	1	1	0	0	2 (4.0)
Could have been prevented						
Yes	4	18	14	5	3	44 (90.0)
No	2	3	0	0	0	5 (10.0)

The responses of the victims after experiencing an episode of violence is described in Table 3. It is important to note that participants who experienced mobbing, sexual harassment and racial discrimination responded in more than one way.

**Table 3:** Response to different types of workplace violence among nursing professionals, Londrina, PR, Brazil, 2018.

Type of violence	Physical	Verbal abuse	Mobbing	Sexual harassment	Racial discrimination	Total n (%)
Response to incident						
No response/pretended it never happened	0	9	11	4	4	28 (57.0)
Told the person to stop	3	7	2	3	0	15 (30.6)
Tried to defend myself physically	2	0	1	1	0	4 (8.0)
Told friends/relatives/supervisor	1	0	0	4	1	6 (12.2)
Requested a transfer	0	1	1	0	0	2 (4.0)
Reported the event	0	4	2	1	0	7 (14.2)

## DISCUSSION

The results showed that most participants were female, demonstrating that nursing is still a predominantly female occupation, associated with the role of a caregiver. It is true that in ancient civilizations, the caretaker role was usually played by women.^15^ As a result, most participants who experienced workplace violence were female, with verbal abuse being the most common type of aggression. Workplace violence is more common in areas predominantly occupied by women, and is associated with sexism and the devaluation of nursing professionals.^17,18^ However, a study of hospitals in northern Portugal revealed that psychological violence was more frequently reported by male nurses (70%), and a review study showed that workplace violence affects all nurses regardless of gender.^19^

In the present study, the fact that most participants were nursing technicians may have contributed to our findings; these workers are more vulnerable to occupational violence, since their close contact with patients increases their exposure to aggression.^20^ As for the frequency of violence, most studies find that these events have occurred once in the previous 12 months, showing that violence is a common feature of the workplace for nurses, with verbal abuse being the most common type of aggression, leading to stress and dissatisfaction with work.^21^ A separate study has also found that workplace violence occurred most frequently in the morning period.^22^

Colleagues and supervisors were the most frequent perpetrators of workplace violence in the present study, followed by patients and their relatives. Our findings also showed that most incidents occurred within the workplace. These results differ from those of another international study involving 219 nurses, which found that patients and their relatives were responsible for most cases of verbal abuse (54.2%), physical violence (47.4%) and sexual harassment (40.9%).^21^ A study of 141 nurses in India also found that verbal abuse perpetrated by patients and their relatives was especially frequent in the sample (98.1%).^21^ A study of 613 nurses and 107 physicians in Macau revealed that the most common forms of workplace violence reported by these individuals were verbal abuse (53.4%), physical aggression (16.1%), bullying (14.2%), sexual harassment (4.6%) and racial discrimination (2.6%). The main perpetrators were patients and their relatives, with colleagues and supervisors accounting for a smaller number of cases; nevertheless, both types of incident had a negative impact on workers’ mental health.^23^ A study in East Africa reported similar findings to the present study, noting that most acts of workplace violence were perpetrated by colleagues.^24^ Furthermore, the study conducted in India found that verbal abuse was reported by 97% of nurses.^21^ This is in line with our findings, which identified verbal abuse and bullying as the most common types of violence reported by nursing staff. Within nursing teams, the most common type of violence is verbal abuse, which is considered less severe than physical aggression.^25^ Verbal abuse was also the most frequently reported type of violence in international studies^21-23^ and one Brazilian study.^9^

Deniz et al.^18^ describe psychological violence as a multidimensional psychosocial syndrome, which affects the individual, their colleagues and the work organization, with both individual and collective effects. Like verbal abuse and mobbing, psychological violence in the workplace is directly associated with depression and burnout syndrome.^25^ Physical violence was not the most frequently cited form of aggression in the present study (11%). This finding is analogous to those of international studies, where physical violence is also found to be less frequent than other forms of abuse.^22,23^

Sexual harassment was also infrequent in the present study; however, a safe work environment should be free from all forms of violence. It is also important to note that in most cases, sexual harassment is ignored or unreported due to embarrassment.^17^ Donne et al.^26^ noted that sexual harassment is underreported due to stigma. Another study found that workers exposed to sexual harassment had elevated scores on all dimensions of burnout syndrome.^27^ Sexual harassment is more common among women, but can also be perpetrated against men, by someone of the same or the opposite gender.^17^ The least frequent form of violence in the present study was racial discrimination. Yet this is by no means a less relevant issue, since invisible racism is a major contributor to social inequality in Brazil.^28^

The notion that violence is an expected part of the work of nurses, and should only be reported when a severe event occurs, leads to a lack of institutional support and hides the magnitude of the problem.^20^ Therefore, not responding to the incident or pretending nothing happened, both of which were common responses in the present study, increases the severity of workplace violence and makes it more difficult to detect. It is crucial for nursing staff to be aware of the different types of violence they may experience in the workplace and that they report these incidents, since they may have a major impact on their health and work-related quality of life.^25^ All forms of violence can have several negative consequences for workers, contributing to increased absenteeism due to psychological issues such as burnout syndrome and minor mental disorders,^12^ and in some cases, even leading to death.^13^ It is important to note that a significant number of participants felt that the violence they experienced could have been avoided. Similarly, an international study found that 82.2% of nurses who experienced occupational violence believed that these incidents could have been prevented.^21^

In most cases, except those involving physical violence, the victims had no response or pretended nothing happened, which may demonstrate the crystallization of violence in these environments or a feeling of impotence in the face of violence and impunity. This is supported by the fact that participants’ reactions were limited to self-defense or asking the perpetrator to stop. It must be noted that every worker is unique, and has a different life history and method of coping with the experience of violence. Nevertheless, workers must be aware of the importance of learning techniques to prevent and reduce violence, since individual-level attitudes can be good for workers, but collective action is ultimately required to address this issue.^13^

In Canada, the Omega program was developed to prevent and manage patient violence against health care workers. The program teaches interpersonal skills and behavioral management strategies that workers can use to intervene in situations of aggression. In the first stage of the program, workers are taught to protect themselves, assess the situation, predict behavior, take the time and focus on the person; in stage two, they are taught a pacification approach to adopt depending on the classification of dangerousness of the behavior; in stage three, workers learn how to adapt their intervention to the behaviors observed; and lastly, in the final stage, they learn how to file post-incident reports.^29^

In the United States, acts of violence in workplace settings prompted nurses to conduct a series of meetings that culminated in the development of an institutional policy to promote a healthy working environment.^30^ The management of workplace violence requires prevention and intervention programs that offer an effective approach to reduce or eliminate risk, using tools such as workplace analysis, preventive measures and health and safety training.^5^ Zero-tolerance policies are also crucial for preventing violence in health care institutions.^23^

Pereira et al.^31^ note the need for local, national and international efforts to implement programs aimed at controlling and preventing workplace violence in nursing settings. This issue cannot continue to go unreported, or used simply as a diagnostic tool. It is crucial that professional organizations and managers join forces to promote better working conditions and, consequently, improve the health of nursing professionals.

The present study had some limitations. As it involved nursing professionals working in a single hospital, the results may not be generalizable to other settings Additionally, responses to the self-report inventories used may have been affected by social desirability or the wish to avoid public exposure. Nevertheless, this study makes important contributions to the discussion and analysis of workplace violence, highlighting the presence of this issue in the lived reality of nursing professionals working in the emergency department of a university hospital. It is crucial that supervisors, together with workers, plan preventive strategies to decrease the incidence of workplace violence, which can be avoided and must be addressed before the lack of active management results in its consolidation as a ‘normal’ and unresolvable issue. Both health promotion and harm prevention measures are needed to provide a safe environment for all workers.

## CONCLUSIONS

The present findings showed that nursing professionals are exposed to several types of violence in their professional practice, including physical violence, verbal abuse, sexual harassment and racial discrimination. Verbal abuse was the most frequent form of violence, and was perpetrated by patients and their relatives, as well as colleagues and supervisors. Female nursing technicians were most affected by workplace violence.

Given the severity with which violence affects workers’ health, and the prevalence of underreporting and stigma, there is a need for workers to be encouraged to identify and report occupational violence, increasing the visibility of the problem and allowing managers and workers to plan and implement strategies to prevent violence and protect workers, in addition to providing a safe working environment with high quality of life.

Prevention/intervention strategies to address violence in hospital settings include: the creation of support networks, monitoring the work environment, implementing procedures for the reporting and referral of severe cases, listening to the attacker and victim, continuing education, encouraging and respecting individual differences and discussing violence in occupational health courses.
